# Chemically Roughened Solid Silver: A Simple, Robust and Broadband SERS Substrate

**DOI:** 10.3390/s16101742

**Published:** 2016-10-19

**Authors:** Shavini Wijesuriya, Krishna Burugapalli, Ruth Mackay, Godwin Chukwuebuka Ajaezi, Wamadeva Balachandran

**Affiliations:** 1 Biomedical Engineering Theme, Institute for Environment, Health and Societies, Brunel University London, Uxbridge UB8 3PH, UK; shav1988@gmail.com (S.W.); Ruth.MacKay@brunel.ac.uk (R.M.); Wamadeva.Balachandran@brunel.ac.uk (W.B.); 2 Department of Mechanical, Aerospace and Civil Engineering, College of Engineering, Design and Physical Sciences, Brunel University London, Uxbridge UB8 3PH, UK; gi14gga@my.brunel.ac.uk; 3 Department of Electronic and Computer Engineering, College of Engineering, Design and Physical Sciences, Brunel University London, Uxbridge UB8 3PH, UK

**Keywords:** SERS substrate, chemical etching, solid silver, surface roughness, 514 nm and 1064 nm Raman

## Abstract

Surface-enhanced Raman spectroscopy (SERS) substrates manufactured using complex nano-patterning techniques have become the norm. However, their cost of manufacture makes them unaffordable to incorporate into most biosensors. The technique shown in this paper is low-cost, reliable and highly sensitive. Chemical etching of solid Ag metal was used to produce simple, yet robust SERS substrates with broadband characteristics. Etching with ammonium hydroxide (NH_4_OH) and nitric acid (HNO_3_) helped obtain roughened Ag SERS substrates. Scanning electron microscopy (SEM) and interferometry were used to visualize and quantify surface roughness. Flattened Ag wires had inherent, but non-uniform roughness having peaks and valleys in the microscale. NH_4_OH treatment removed dirt and smoothened the surface, while HNO_3_ treatment produced a flake-like morphology with visibly more surface roughness features on Ag metal. SERS efficacy was tested using 4-methylbenzenethiol (MBT). The best SERS enhancement for 1 mM MBT was observed for Ag metal etched for 30 s in NH_4_OH followed by 10 s in HNO_3_. Further, MBT could be quantified with detection limits of 1 pM and 100 µM, respectively, using 514 nm and 1064 nm Raman spectrometers. Thus, a rapid and less energy intensive method for producing solid Ag SERS substrate and its efficacy in analyte sensing was demonstrated.

## 1. Introduction

Surface enhanced Raman spectroscopy (SERS) has a great potential for analytical testing due to:

- the high sensitivity and selectivity, including the possibility of single molecule detection;
- the possibility of multiplexed analysis;
- the availability of cheaper and better hand held Raman spectrometers; and
- the potential of point-of-sampling testing, including at home, in the clinician’s office, and in the field involving standalone battery operated portable analytical instrumentation [1,2,3,4,5,6,7,8,9,10].

However, SERS is yet to reach routine analytical use [3]. The major bottlenecks for routine use of SERS include reliability, cost and the short shelf-life of the SERS substrates [1,2,3,4,5]. There have been several SERS substrate designs developed through three decades of research, but most substrate designs fall short of one or more of the above-mentioned bottlenecks [2,3,4]. SERS substrate designs broadly fall under three categories:

1. electrochemically roughened metal electrodes;
2. metal colloids; and
3. engineered metallic nano-patterns on planar surfaces [2].

Electrochemically roughened Ag electrodes were the first substrates on which the SERS phenomenon was discovered [11]. They are relatively low-cost to produce, but have poor SERS efficiency [1,2]. Metal colloids, on the other hand, are known to provide the best SERS enhancements [2]. However, their SERS activity is dependent on the aggregation of metal nanoparticles (NPs) with optimum distance between the NPs, which is difficult to reproduce consistently [3]. The third design category allows precise control of nano-patterns, thus producing SERS substrates with reproducible uniformity and SERS efficiency [1,2]. This precision engineering involves tedious methods and sophisticated instrumentation, making the production of nano-patterned SERS substrates expensive [1,2]. The need for specialist expertise and fabrication equipment in the design of SERS substrates, especially for the latter two design categories, has meant SERS substrates are unaffordable for routine use [1] As a result, there is a need for simple SERS substrates that are inexpensive and easy to produce [1].

A simple, fast, and inexpensive method for generating effective SERS substrates is the traditional wet chemical etching of a solid metal. The method involves immersion of the metal in an etching solution for a few seconds to minutes depending on the strength of the etching agent. The etching chemistry is typically an electrochemical process caused by the oxidation of the metal surface. An etching agent, an acid or a base, acts as a redox agent that corrodes the metal surface, while its diluent medium, e.g., water, removes the reactants and products, usually resulting in a clean surface [12,13]. If the etching is strong enough, rough nano-topography essential for SERS is generated on the metal surface. Pronounced SERS signals were reported for solid Cu and Ag foils etched with concentrated nitric acid [14,15,16,17,18,19].

It is now widely accepted that an intense electromagnetic field is generated by incident light on a plasmonic surface (typically of a noble metal, e.g., Ag, Au or Cu), which is the chief contributor to SERS enhancement, and potentially any material capable of generating the plasmonic field can generate a SERS signal [20]. In practice, Ag and Au metals are commonly used because they are stable in air (oxygen) and (pure) water [2]. Between Ag and Au, Ag produces the largest SERS enhancement (better SERS efficacy), while Au is more stable (longer shelf-life) and has better biocompatibility. However, the actual material choice depends on several factors including the surface plasmon resonance wavelength and the surface nano-topography of the plasmonic material, the excitation wavelength, the spectroscopic properties of the molecule (analyte) to be tested, and the experimental setup [2,21,22,23]. In essence, to maximize the SERS efficiency, there would be a need to identify the right combination of the metal, its nano-structure, excitation wavelength and experimental setup for each test analyte [24]. However, it would be practical to have a universal SERS substrate that can provide meaningful SERS enhancement for detection and quantitation of any Raman active analyte of interest.

From a practical perspective, chemically roughened solid Ag metal could be one of the closest to a simple and universal SERS substrate. Ag is known for its broad plasmon resonant band, ranging from visible to near infra-red region and a large imaginary part of dielectric constants and, hence, Ag-based SERS substrates, in general, provide the most effective SERS surface [2]. Chemically roughened solid Ag metal has further benefits [16]:

- they are simpler, faster, and cheaper to produce than electrochemical roughening, metal colloids or nano-structure arrays;
- avoiding salts in the chemical etching solution, unlike the electrochemical roughening process, produces clean Ag surfaces;
- there is no need for costly instrumentation for their production;
- Ag surfaces may suppress the undesired interference from florescence; and
- Ag, being an excellent conductor of heat, provides good thermal stability to the chemically roughened solid SERS substrate, and hence, would minimize the sample decomposition due to the heat generated by the laser irradiation, especially, by the high power lasers required for longer wavelength Raman spectroscopy.

The objective for this study was to evaluate simple chemical etching methods to generate roughened solid Ag surfaces for SERS, and identify one such method that provides a reproducible surface roughening (hotspots) and better SERS sensitivity. Specifically, we evaluated the effects of chemical etching using ammonium hydroxide (NH_4_OH), nitric acid (HNO_3_) and/or heat treatment on the roughening of flattened Ag wires. The resulting wires were characterized for their roughness using scanning electron microscope (SEM) and interferometry, surface composition using Energy-dispersive X-ray Spectroscopy (EDS), and SERS efficacy using 1 mM 4-methyl benzene thiol (MBT) as a model Raman active molecule and Raman spectrometers equipped with 514 nm and 1064 nm lasers for recording SERS spectra. The choice of the 514 and 1064 nm laser Raman spectrometers for SERS efficacy testing was to demonstrate the broad band characteristic of the Ag metal’s surface plasmon resonance.

## 2. Materials and Methods

Silver wire, 99.99% (Ag10T) 0.25 mm diameter with a Teflon coating was purchased from MedWire, Sigmund Cohn (Mount Vernon, NY, USA); 18 mΩ deionised (DI) water purified using a Barnstead water purification system (Waltham, MA, USA) was used for all experiments; NH_4_OH solution (35%), HNO_3_ (≥69%), and 4-Methylbenzenethiol (98%) (MBT) were purchased from Sigma-Aldrich Ltd. (Gillingham, Dorset, UK); and absolute ethanol was purchased from Fisher Scientific Ltd. (Loughborough, Leicestershire, UK).

To fabricate roughened solid Ag metal SERS substrates, 1.5 cm long Ag wire segments were cut and their Teflon cover removed. The wires were then manually flattened using a mechanical press. To wash off surface contaminants the flattened wires were ultra-sonicated in DI water for 30 min using a Branson 1510 sonicator. The surface of the flattened wires was then roughened using different chemical etching treatments: passive immersion in:

1. 35% NH_4_OH for 30 s;
2. 6 M HNO_3_ for 10 s;
3. 6 M HNO_3_ for 2 min;
4. 35% NH_4_OH for 30 s followed by 6 M HNO_3_ for 10 s; and
5. 35% NH_4_OH for 30 s followed by 6 M HNO_3_ for 10 s.

Following immersion in NH_4_OH or HNO_3_, the wires were rinsed in deionised water. The roughened Ag wires were then air-dried at room temperature. In addition, the effect of heat treatment, 130 °C for 1 h, on the roughness and SERS efficacy of the different chemically roughened Ag wires was also tested.

The effect of chemical treatments ± heat treatments on roughness and composition of the Ag metal surface was characterised using the Benchtop JCM-6000 NeoScope SEM equipped with EDS (JEOL, Welwyn Garden City, Hatfield, UK). Interferometry (Zygo New View 5000 (AMETEK Germany GmbH, Weiterstadt, Germany) equipped with MetroPro software) was also used to visualise and quantify the surface roughness on the different Ag substrates. The roughness parameters quantified using Zygo interferometer include R_a_ (arithmetic mean roughness), R_t_ (sum of the maximum peak height and maximum valley depth), R_p_ (maximum peak height), R_v_ (maximum valley depth) and R_q_ (root mean square roughness) of the surface area used for measurements [25].

Raman spectra for the different Ag substrates were recorded using two different Raman spectrometers. First was a benchtop Renishaw InVia confocal Raman microscope (Wotton-under-Edge, Gloucestershire, UK) equipped with 514 nm laser having maximum power output of 50 mW. The samples were scanned with a 20 × 0.4 NA objective, 12.68 µm laser spot size, 10% power, 10 s acquisition time, 4 cm^−1^ resolution and spectra recorded using Wires 3.3 software. Second was a portable StellarNet Inc. (Tampa, FL, USA) 1064 nm Raman spectrometer with diode laser having maximum power output of 499 mW. The samples were scanned using a fibre optic probe at a working distance of 7.5 mm, 158 µm laser spot size, ~250 mW laser power, 5000 ms acquisition time, 8 cm^−1^ resolution and spectra recorded using SpectraWiz software. Cubic spline interpolation tool on Wires 3.3 software was used for the baseline corrections of the spectra recorded using both 514 nm and 1064 nm Raman spectrometers.

To determine their SERS efficacy, the different test Ag substrates were immersed in 1 mM MBT solution in ethanol for 24 h. The substrates were then rinsed three times in ethanol and air dried before scanning for Raman spectra. Solid MBT was also scanned for control Raman spectrum. The substrate that showed the best SERS enhancement namely Ag substrate etched for 30 s in NH_4_OH followed by 10 s in HNO_3_ was then further tested for efficacy in quantitation of MBT.

Statistical differences between groups (*p* < 0.05) were determined by one-way analysis of variance (ANOVA) using SPSS v20. Tukey’s significant difference test was used for post hoc evaluation.

## 3. Results and Discussion

Betz et al. [1] identified four characteristics for fabrication of simple SERS substrate:

1. fabricated using equipment and reagents commonly found in chemistry laboratories;
2. minimal training or experience required for fabrication;
3. easily transported to or fabricated at the point of sampling; and
4. easily integrated into analytical systems.

The passive chemical etching method shown in this study is the closest to a simple SERS substrate. It uses common and inexpensive laboratory chemicals—NH_4_OH and HNO_3_—for generation of simple SERS substrate on solid Ag metal surface. The chemical roughening process involves simple passive immersion in NH_4_OH and HNO_3_ for a few seconds, which would require minimal training or experience. An added advantage for this method is the possibility of regeneration of the Ag metal surface by further passive chemical etching treatment(s). The substrate can potentially be fabricated at the point of sampling in a matter of minutes. It can also be easily integrated on a point-of-care device and scanned for SERS spectrum using a portable Raman spectrometer at the point of sampling. Furthermore, to our knowledge, the synergistic effect of NH_4_OH and HNO_3_ in the generation of roughened Ag metal based SERS substrate has not been reported earlier.

Nitric acid etching has been shown to produce roughened solid metal surfaces for SERS [14,15,16,17,18,19]. Miller et al. used 2 mole/dm^3^ HNO_3_ or a combination of sandblasting and etching to obtain surface roughness features on the 10–100 nm scale on Cu surface [14]. These substrates were shown to provide SERS enhancements of the order of 10^3^ to 10^4^ and 10^5^ to 10^6^, respectively, for Nile Blue and p-nitrobenzoic acid using a 662 nm laser Raman spectrometer [14]. Xue and Dong showed the efficacy of HNO_3_ etching in generating simple roughened Ag metal SERS substrates [16]. Unlike for Cu surface, Xue and Dong reported that a higher concentration of HNO_3_ is needed for roughening of Ag surface. They immersed and vigorously stirred Ag foils for 2–3 min in 3.5 M HNO_3_ at ambient temperature to create a sponge-type surface with abundant roughness features on a 10–100 nm scale [16]. Xue and Dong reported this SERS substrate to provide stable SERS enhancements in various solvent media and elevated temperatures making them useful in studies of adsorption, molecular orientation, surface reaction, and surface catalysis at room or elevated temperatures [16]. Laserna et al. also reiterate that nitric acid etched Ag metal exhibits a strong SERS effect and excellent stability, reversibility and spectral response [17,18,19].

In our lab, we routinely use etching of Ag wires with 30 s 35% NH_4_OH followed by 10 s in 6 M HNO_3_ to obtain a rough surface (increased surface area) for making Ag/AgCl electrode for electrochemical sensing [26,27,28]. In this study, we investigated the efficacy of NH_4_OH and HNO_3_ in the chemical etching of the Ag metal for generating roughened solid Ag SERS substrates. NH_4_OH is a weak base that is commonly used for household cleaning and polishing of jewellery, wherein, it is typically used at concentrations of 5% to 10%. This generally lifts off any dirt and grime from the surface. However, when used at higher concentrations and longer immersion durations for cleaning silver metal, aqueous NH_4_OH is known to cause surface etching. For SERS, roughness features of 10 to 100 nm and their optimum spacing on a surface is desired [14,15,16,17,18,19]. Therefore, the use of the weak base NH_4_OH at a higher concentration (35%), we envisioned would produce a roughened surface on the flattened Ag wire. On the other hand, HNO_3_ is a strong acid that aggressively dissolves Ag metal that could generate rough surface with lesser time of treatment. Thus, depending on the nature of etching solution and the length of the etching process, the roughness generated on the metal surface varies.

**Figure 1:**
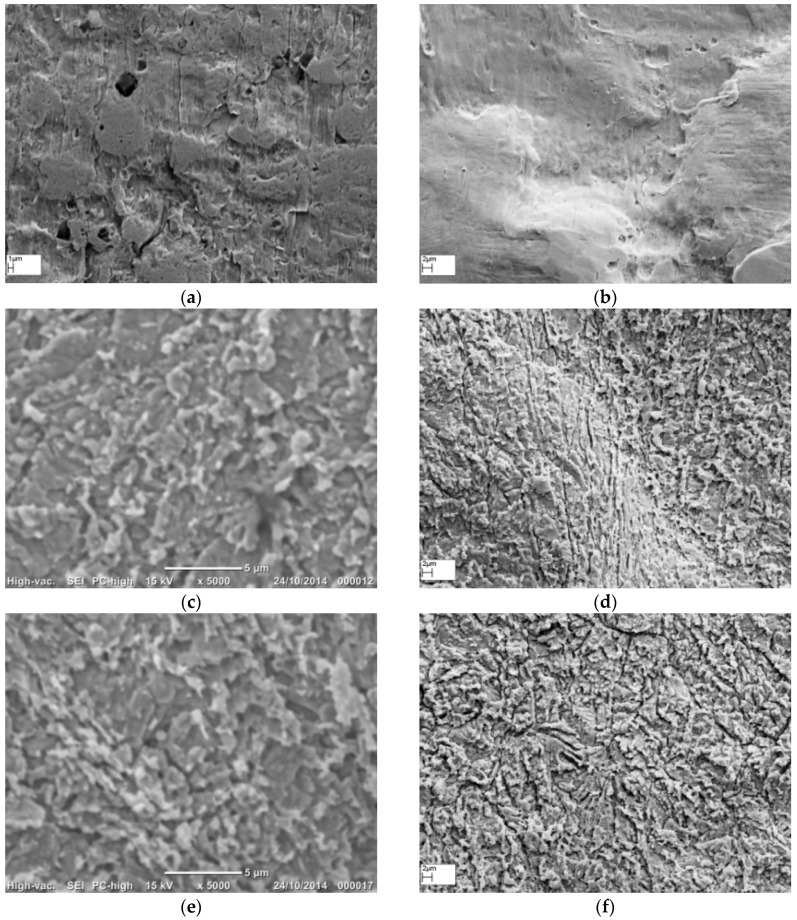
Effect of the different chemical etching treatments on surface morphology of Ag metal surface as seen under scanning electron microscope (SEM): (**a**) native as-flattened Ag wire; (**b**) 30 s NH_4_OH; (**c**) 10 s HNO_3_; (**d**) 2 m HNO_3_; (**e**) 30 s NH_4_OH followed by 10 s HNO_3_; and (**f**) 30 s NH_4_OH followed by 2 m HNO_3_.

To identify an optimum treatment, we investigated five different treatment regimens: (1) 30 s NH_4_OH; (2) 10 s HNO_3_; (3) 2 m HNO_3_; (4) 30 s NH_4_OH followed by 10 s HNO_3_; and (5) 30 s NH_4_OH followed by 2 m HNO_3_. To our knowledge, the use of NH_4_OH on its own for the generation of roughened Ag metal as SERS substrate has not been reported earlier. However, NH_4_OH in combination with H_2_O_2_ was reported by the group of Innocenti, and by Goh et al. [29,30,31] It is remarkable to see similar morphology on Ag metal surface treated with NH_4_OH + H_2_O_2_ as reported by Innocenti et al. [29] to that we saw in our study with NH_4_OH treatment on its own (Figure 1b). As discussed in the following sections, NH_4_OH being a weak base showed slow etching (even with high concentration (35%)) lifting off a uniform and thin layer of Ag from the surface of the Ag metal, resulting in surface roughness features more or less similar to non-treated original Ag metal surface. Hence, for this study we limited the treatment with NH_4_OH on its own to just one concentration. On the other hand, HNO_3_ is a strong acid that rapidly etches and dissolves Ag metal, and as discussed in the following sections, HNO_3_ did produce the rough surface desirable for a SERS substrate on Ag metal. We limited the HNO_3_ treatments to two time points 10 s and 2 m, since the longer we immerse in HNO_3_, the more Ag dissolves and gets wasted. This is detrimental to the idea of a simple SERS substrate. Furthermore, we were pleasantly surprised to find the best SERS performance with 30 s NH_4_OH followed by 10 s HNO_3_ treatment, thus minimizing the wastage of Ag metal in this optimised SERS substrate fabrication method. However, the effect of longer and more vigorous HNO_3_ treatments on SERS efficacy of roughened Ag metal was reported by Perez et al. [19]. They tested 1 to 5 m vigorous stirring of Ag foils in 6 M HNO_3_, and noted that the SERS efficacy decreases with increasing etching time, and can result in complete dissolution of the Ag foil. Our report here provides further insight (based on the interferometry results) that it is not only the spacing between neighbouring surface roughness features (generated by HNO_3_ treatment), but also their height that effect the SERS performance of the roughened silver metal SERS substrates. Moreover, the synergistic etching with NH_4_OH followed by HNO_3_ that we report here speeds up the etching, without the need for vigorous stirring in the method reported by Xue and Dong, and Laserna et al. [14,15,16,17,18,19]. Thus, our method avoids the need for any energy intensive instrumentation for the fabrication of the Ag metal SERS substrate.

### 3.1. Morphology of Roughened Ag Substrates as Visualised Under SEM

The 99.99% pure Ag wire of 0.25 mm diameter was used as the solid Ag metal for this study. The wire was flattened using a mechanical press to obtain a flat surface for Raman spectroscopy. The flattening process using a mechanical press introduced some debris (dark spots) on the Ag wire surface (Figure 1a). However, when this wire was treated with 35% NH_4_OH for 30 s, its surface changed into a bright, shiny and reflective silver colour to the naked eye. Under SEM, the NH_4_OH treated wire appeared clean and had a smoother surface (Figure 1b) compared to the original flattened Ag wire (Figure 1a). The surface of the 10 s HNO_3_ treated Ag wire appeared flaky, with narrow gaps in between the flaky structures that are numerous compared to the rough features observed on the native as well as the NH_4_OH treated Ag wires (Figure 1c). On the 2 m HNO_3_ treated surface, the flakes were worn out into smaller separated protrusions that appear like elevated peaks (Figure 1d).

Thereafter, we studied the synergistic effect of surface cleansing and smoothening caused by NH_4_OH treatment and the uniform etching caused by HNO_3_ treatment on the surface features and roughness on Ag wires. The morphology and topography of the 30 s NH_4_OH + 10 s HNO_3_, and 30 s NH_4_OH + 2 m HNO_3_ treated Ag wires is shown in Figure 1e,f. The combination of NH_4_OH followed by 10 s or 2 m HNO_3_, resulted in similar morphology to that observed with the respective 10 s and 2 m HNO_3_ treatments, except that the valleys between the flaky or discrete features, respectively, appeared wider and deeper. This adds variety to the surface features on the Ag wires, which could potentially provide varying degrees of SERS enhancements.

**Figure 2:**
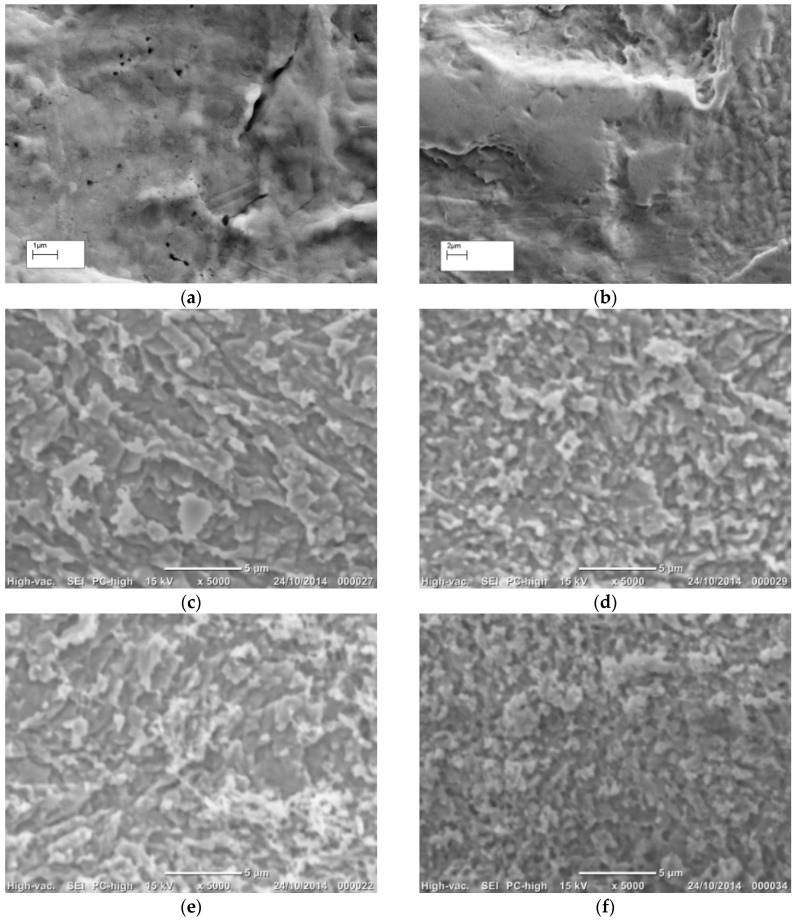
Effect of heat treatment, at 130 °C for 1 h, on surface morphology of the different chemical etched Ag surfaces: (**a**) native flattened Ag wire washed in DI water under sonication; (**b**) 30 s NH_4_OH; (**c**) 10 s HNO_3_; (**d**) 2 m HNO_3_; (**e**) 30 s NH_4_OH followed by 10 s HNO_3_; and (**f**) 30 s NH_4_OH followed by 2 m HNO_3_.

Heat treatments were reported to affect the SERS efficacy of roughened metal surfaces [16]. Hence, we utilised heat treatment at 130 °C for 1 h to evaluate its physical and chemical influence on the surface properties of the native and the chemically etched Ag wires [16]. The SEM images of the heat treated native (Figure 2a), 30 s NH_4_OH (Figure 2b), 10 s HNO_3_ (Figure 2c), 2 m HNO_3_ (Figure 2d), 30 s NH_4_OH + 10 s HNO_3_ (Figure 2e), and 30 s NH_4_OH + 2 m HNO_3_ (Figure 2f) Ag substrates all showed morphology comparable to their non-heated counter parts (Figure 1a–f).

### 3.2. Topography of Roughened Ag Substrates as Mapped Using Zygo Interferometer

**Figure 3:**
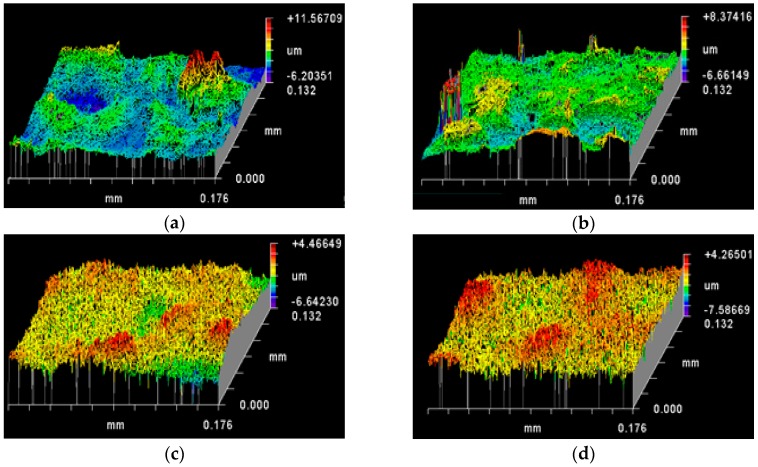

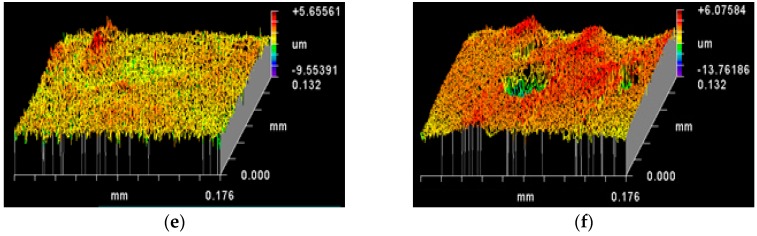
Effect of chemical etching treatments on surface topography (as mapped by Zygo interferometer): (**a**) native flattened Ag wire; (**b**) 30 s NH_4_OH; (**c**) 10 s HNO_3_; (**d**) 2 m HNO_3_; (**e**) 30 s NH_4_OH followed by 10 s HNO_3_; and (**f**) 30 s NH_4_OH followed by 2 m HNO_3_ etching treatments.

The topography data presented here are the 3D surface maps recorded using Zygo New View 5000 interferometer equipped with MetroPro software. The quantitative data, namely, roughness parameters, associated with these 3D surface maps represent the non-periodic finer irregularities in the surface texture that provide a measure of the vertical characteristics of the surface [25]. Roughness parameters are generally abbreviated with R followed by letters in subscript, and are calculated on a profile (line) or on a surface (area) [25]. R_t_ is the sum of the maximum peak height (R_p_) and the maximum valley depth (R_v_) as measured from the mean linear surface on the Ag wire surface. R_a_ is the arithmetic mean roughness, while R_q_ is root mean square roughness. The difference between R_a_ and R_q_ indicates the uniformity in roughness, since R_q_ is more weighted by large values of peak height and valley depth [32]. The RMS value is typically 10%–25% larger than the mean value of roughness depending on the nature of the surface [32]. The surface topography profile maps and associated roughness parameter data for the different chemical etched Ag substrates are presented in Figure 3 and Table 1, respectively.

The surface map (Figure 3a) and the SEM image (Figure 1a) show that the flattened native Ag wires have an inherently rough topography with plateaus (green and yellow colours), troughs (blue colour indicating valleys) and irregular features (red colour indicating peaks/summits). The roughness of flattened native Ag wires was in the microscale with an R_t_ value of 11.15 ± 0.89 µm and an R_a_ of 0.95 ± 0.10 µm. The R_a_ lesser than 1 and the more green colour compared to yellow in the surface topography maps could indicate that the mean linear surface of the Ag wires is skewed towards the valleys (more average valleys compared to the average peaks across the surface profile) (Figure 1c and Table 1). However, a 32.20% higher R_q_ value compared to its corresponding R_a_ indicates that there are more high peaks and deep valleys deviating from mean linear surface of the Ag wires (Table 1) leading to an overall rougher surface.

NH_4_OH has a caustic effect on Ag surface, wherein it lifts off any dirt, loose particles as well as Ag metal from the surface. In this process, NH_4_OH treatment not only removed any debris, but also etched a layer of Ag metal on the inherently rough flattened Ag wire surface. This etching is reflected by the decrease in roughness parameters R_q_ (1.02 ± 0.19 µm vs. 1.27 ± 0.17 µm) and R_a_ (0.77 ± 0.15 µm vs. 0.95 ± 0.10 µm), without significant difference in the percentage increase in R_q_ vs. R_a_ (31.90% vs. 32.20%)) for NH_4_OH treated Ag wires compared to that for the native Ag wires (Table 1 and Figure 3b). However, R_a_ was significantly lower than 1 for the NH_4_OH treated Ag wires, which could indicate that the mean linear surface is acutely skewed towards the valleys. Thus, the NH_4_OH treatments appear to wear the surface peaks faster than the valleys.

Unlike NH_4_OH, HNO_3_ is a strong acid that rapidly dissolves Ag metal. In this study, we used 6 M aqueous HNO_3_ for passive etching the surface of the flattened Ag wires and the etching durations were either 10 s or 2 m. The quantitative topography data further reflected the differences in the surface roughness on the Ag wires induced by HNO_3_ treatments, when compared to that observed on the native and NH_4_OH treated Ag wire surfaces (Table 1 and Figure 3c,d). For both 10 s and 2 m HNO_3_ treatments, the R_q_ and R_a_ values were not significantly different with that observed on the native Ag wires. However, a lower per cent increase in R_q_ vs. R_a_ for both 10 s and 2 m HNO_3_ treated surfaces compared to native Ag wire surface indicate that there are now lesser number of high peaks and deep valleys deviating from mean linear surface on the HNO_3_ surfaces making their roughness relatively uniform. The R_a_ values close to 1 further indicate that there is a balance between the number of peaks and the number of valleys on the HNO_3_ treated Ag wire surfaces. Thus, the stronger HNO_3_ solutions appeared to cause uniform etching, when compared to NH_4_OH treatment that caused faster etching of peaks vs. valleys. In addition, there appear to be more surface features that are uniformly distributed on the surface, which is a desired surface for SERS signal enhancements. Between the 10 s and 2 m HNO_3_ treatments, there is significant difference in the features on the surface as discussed above. It is the anisotropic shapes and separation of these surface feature, that determine the degree of SERS enhancement, and hence we have two interesting surfaces with different surface features, but similar roughness between the 10 s and 2 m HNO_3_ treated Ag wires.

The 30 s NH_4_OH + 10 s HNO_3_ treated Ag wires showed an R_a_ value of 1.05 ± 0.21 µm and per cent increase in R_q_ vs. R_a_ of 33.52%, slightly higher than that observed with the native Ag wires (0.95 ± 0.10 µm), skewing the mean linear surface towards the peaks (Table 1 and Figure 3c). However, the higher R_q_ (1.40 ± 0.30 µm) indicates that there are more high peaks and low valleys away from the mean linear surface, which in turn is suggestive of more surface features per unit area on the 30 s NH_4_OH + 10 s HNO_3_ treated Ag wires compared to the native Ag wires (Table 1 and Figure 3c).

On the other hand, 30 s NH_4_OH + 2 m HNO_3_ treated Ag wires had significantly higher R_a_, R_q_, and per cent increase in R_q_ vs. R_a_, suggesting the roughest surface among all the chemical etching treatments in this study (Table 1 and Figure 3f). The reference mean linear surface is significantly skewed towards peaks (R_a_ of 1.39 ± 0.30 µm). There are more number of high peaks and deep valleys away from the mean linear surface (R_q_ of 1.95 ± 0.41 µm). It also has the significantly rougher surface (40.08% higher R_q_ vs. R_a_) among all the chemical treatments tested in this study (Table 1 and Figure 3f).

**Figure 4:**
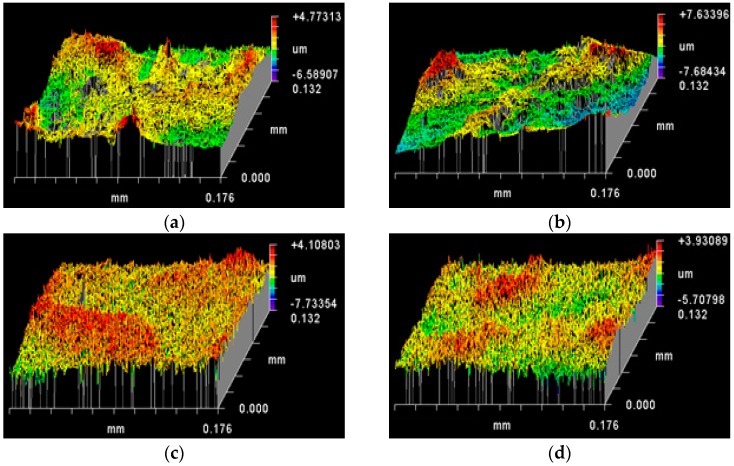

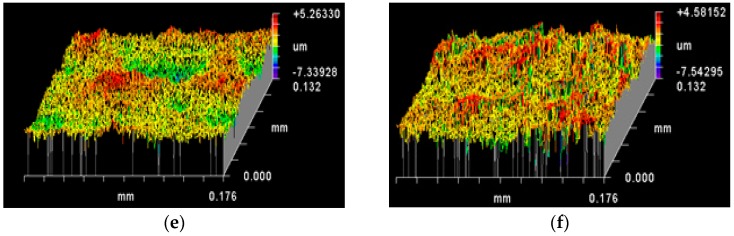
Effect of heat treatment at 130 °C for 1 h on the surface topography of the different chemical etched Ag substrates (as mapped by Zygo interferometer): (**a**) native flattened Ag wire; (**b**) 30 s NH_4_OH; (**c**) 10 s HNO_3_; (**d**) 2 m HNO_3_; (**e**) 30 s NH_4_OH followed by 10 s HNO_3_; and (**f**) 30 s NH_4_OH followed by 2 m HNO_3_ etching treatments.

Heating at 130 °C for 30 m did not appear to cause significant difference in surface morphology on SEM images to naked eye (Figure 1 and Figure 2). However, quantitative topography results showed differences in surface roughness parameters between the heated (Figure 4, Table 2) and their non-heated chemical roughened Ag substrate counterparts (Figure 3, Table 1). Ag is a soft and malleable metal, and the changes in the roughness parameter R_a_, R_q_ and per cent increase in R_q_ vs. R_a_ for all Ag surface variables, in this study, were suggestive that heating at 130 °C for 1 h induced restructuring of surface features on the Ag wires into less rougher surfaces compared to their respective non-heated counterparts. The per cent increase in R_q_ vs. R_a_ for the different heated Ag wires ranged between 26% and 33%, which was lower compared to their non-heated counterparts (27% to 40%). This indicates that the number of peaks higher and valleys deeper than the mean linear surface reduced. However, for 30 s NH_4_OH treatment, heat treatment resulted in an increased R_a_ and R_q_ values compared to its non-heated counterpart. This was in contrast to all of the other chemical etching treatments, where heating reduced the R_a_ and R_q_ values. One explanation for this could be that, the NH_4_OH treatment could leave amine residues on the Ag surface, since Ag metal is known to passively react with amines. NH_4_OH residues on a metal, when heated, are known to cause the nitriding effect, wherein the NH_4_OH dissociates into N_2_ and H_2_. N_2_ diffuses onto the metal surface creating a nitride layer that is known to increase the mechanical properties and roughness of the surface [33].

### 3.3. Surface Chemical Composition as a Function of Etching Treatments

**Figure 5:**
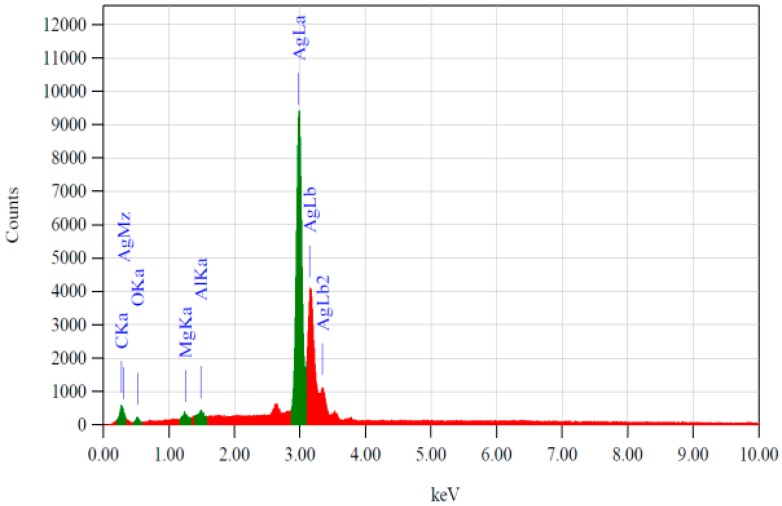
A typical energy-dispersive X-ray (EDS) spectrum of the flattened Ag metal predominantly showing peaks for Ag metal: AgLa, AgLb and AgLb2 lines at 2.983, 3.151 and 3.348 keV, respectively. Traces of C, O, Mg and Al were also seen at 0.277, 0.525, 1.253, 1.486 keV, respectively. The spectra were similar for the different test samples in this study, irrespective of the chemical etching treatments with and without heating.

SERS, being a sensitive transduction mechanism, requires a clean metal surface before it is treated with an analyte of interest for detection or assay. Any contaminant on the SERS substrate surface could give unwanted SERS spectral peaks. Hence, it was important to evaluate the chemical composition, which was accomplished by recording EDS spectra. Figure 5 shows an example of the EDS spectrum as observed for the flattened Ag metal treated for 30 s with NH_4_OH. The primary peaks observed were that of Ag (indicating high purity, >99%). A very small peak for carbon and occasional peaks for oxygen, magnesium and aluminium were also observed, indicating the presence of residual hydrocarbons and trace amounts of metals (Mg, Al). Interestingly, irrespective of the chemical treatment performed, in this study, to etch the Ag wires as well as of the heating, all the silver surfaces tested showed EDX spectra similar to that shown in Figure 5.

### 3.4. Efficacy of the Roughened Ag Wires in Enhancing SERS Signal

**Figure 6:**
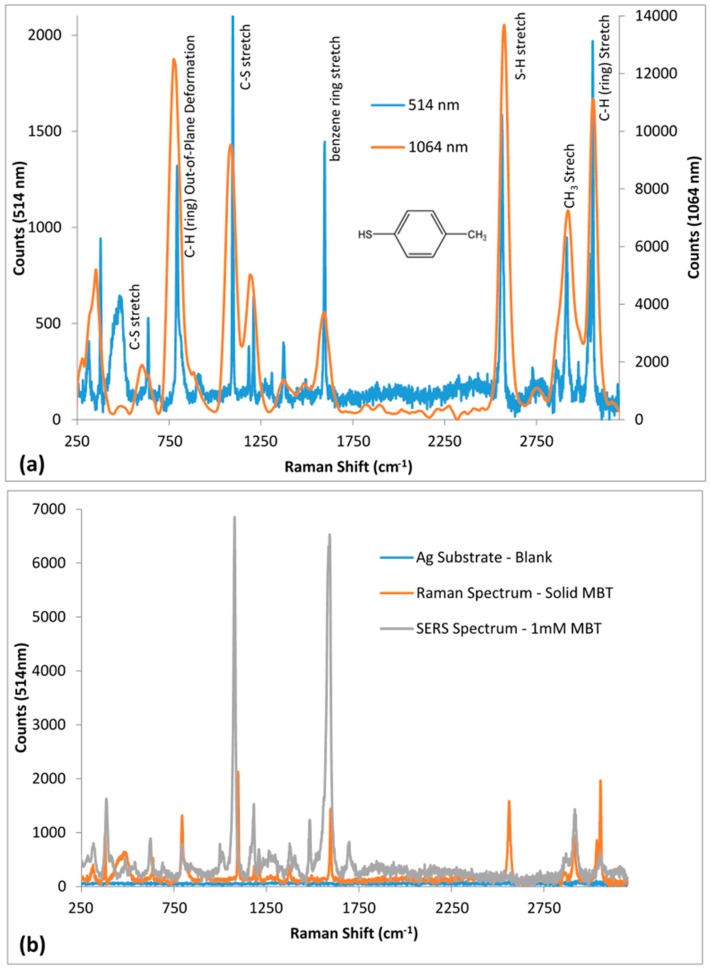

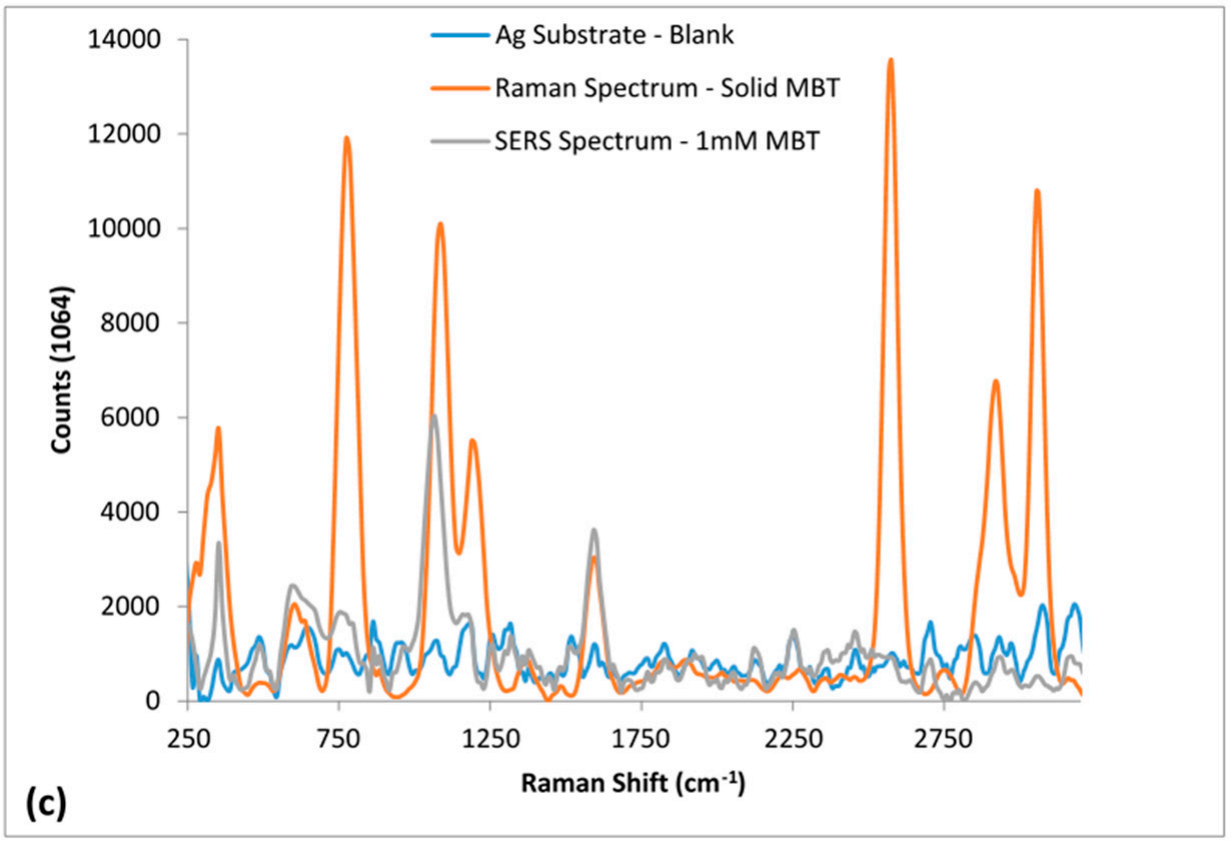
Raman and SERS spectra of MBT recorded using 514 nm and 1064 nm Raman spectrometers: (**a**) 514 nm and 1064 nm Raman spectra of solid MBT; and (**b**,**c**) 514 nm and 1064 nm SERS spectra respectively, for 1 mM MBT on 30 s NH_4_OH + 10 s HNO_3_ Ag substrate compared to Raman spectrum of solid MBT and the base Ag substrate (blank).

MBT, also known as p-thiocresol, is a commonly used Raman probe molecule for SERS efficacy testing [34]. In this study, we tested the SERS efficacy of the different chemically etched Ag substrates, both heated and non-heated using MBT. The Raman spectra for solid MBT, recorded using 514 nm and 1064 nm Raman spectrometers are shown in Figure 6a. The prominent Raman peaks (514 nm; 1064 nm) of interest observed for bulk MBT include: S–H stretch (2562 cm^−1^; 2573 cm^−1^), benzene ring stretch (1591 cm^−1^; 1592 cm^−1^), C–S stretch (1097 cm^−1^ and 634 cm^−1^; 1085 cm^−1^ and 609 cm^−1^), C–H torsion for benzene ring (794 cm^−1^; 781 cm^−1^), C–H stretch for CH_3_ group (2914 cm^−1^; 2918 cm^−1^) and C–H stretch for benzene ring (3036 cm^−1^ and 3056 cm^−1^; 3059 cm^−1^) [34,35,36,37,38,39,40].

For SERS efficacy testing, the Ag substrates were immersed overnight in 1 mM MBT in ethanol. They were then washed 3 times with ethanol, to remove any non-bound MBT. For each test substrate the SERS spectra were recorded using both 514 nm and 1064 nm Raman. A comparison of the SERS spectrum of 1 mM MBT with that of the corresponding Raman spectrum of the bulk MBT (98%) recorded for 30 s NH_4_OH + 10 s HNO_3_ Ag substrate using 514 nm and 1064 nm Raman are illustrated in Figure 6b,c, respectively. Further, the corresponding Raman/SERS peaks are listed in Table 3.

**Figure 7:**
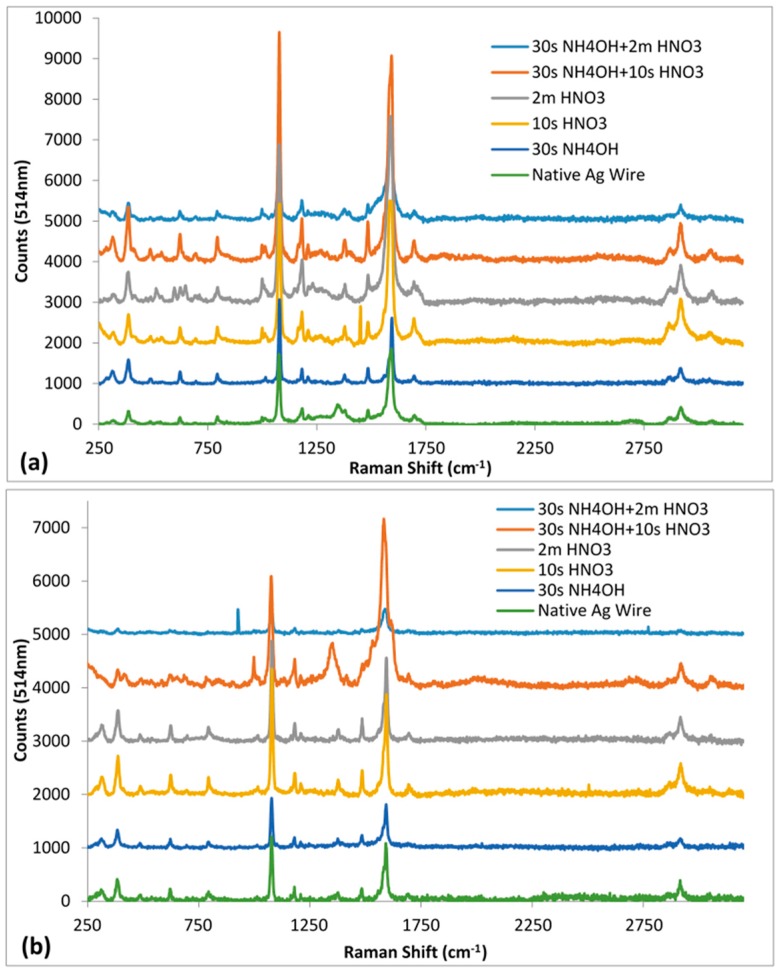
The 514 nm Raman spectra for the native and chemically treated Ag wires with 1 mM MBT: (**a**) non-heated; and (**b**) heated. Spectra offset for clarity.

The absence of the S–H stretch peaks at 2562 cm^−1^ and 2573 cm^−1^, respectively, in the 514 nm and 1064 nm SERS spectra shows that all MBT molecules are bound on the silver surface through Ag–S covalent bonding (Figure 7a,b). Two prominent SERS peaks reported in literature for MBT are approximately at 1075 cm^−1^ and 1590 cm^−1^ [34]. Both these peaks are prominently enhanced in both the 514 nm (1076 cm^−1^ and 1584 cm^−1^) and 1064 nm (1063 cm^−1^ and 1592 cm^−1^) SERS spectra, as shown in Figure 6b,c, respectively. As expected, SERS spectrum for 1 mM MBT was strong and detailed for 514 nm compared to 1064 nm. The 514 nm SERS spectrum for MBT showed majority of the peaks seen in the Raman spectrum for solid MBT (Table 3): 314, 388, 623, 791, 1076, 1180, 1377, 1483, 1584, and 2919 cm^−1^ on SERS spectrum corresponding to 312, 377, 637, 794, 1097, 1184, 1374, 1492, 1591 and 2914 cm^−1^, respectively, on Raman spectrum. The slight shift in peaks can be attributed to the changes in chemical environment of MBT on Ag surface.

On the other hand, there were fewer peaks in the 1064 nm SERS spectrum for MBT (353, 608, 1063, and 1592 cm^−1^) compared to the 1064 nm Raman spectrum for solid MBT (347, 609, 781, 1085, 1198, 1372, 1592, 2573, 2739, 2918 and 3059 cm^−1^) (Table 3). This can be attributed to the weaker SERS signal due to the longer 1064 nm laser (inverse relation of SERS signal to the fourth power of the laser wavelength).

The 514 nm SERS spectra for both non-heated and heated chemical etched silver substrates showed spectra resembling that observed for solid MBT (Figure 7a,b). Two SERS peaks of interest for MBT that are prominently enhanced are ~1076 cm^−1^ and ~1595 cm^−1^. The absolute intensities for these two SERS peaks for MBT were compared to determine the SERS efficacy of the different test Ag substrates in this study. The SERS spectral data in conjunction with the interferometry based surface roughness results indicated that the separation between surface roughness features plays an important role in determining the degree of SERS enhancements (Figure 7a,b, Table 4).

The enhancement factor (EF) for the SERS peaks, ~1076 cm^−1^ and ~1595 cm^−1^, of MBT was estimated using the standard equation: EF = (I_SERS_/N_SERS_)/(I_RS_/N_RS_), where I_SERS_ was the SERS absorption counts (absolute intensity) provided by the number of MBT molecules (N_SERS_) estimated to be present within the incident laser light field, while I_RS_ was the corresponding absolute intensity provided by the estimated number of MBT molecules (N_RS_) in the normal Raman spectrum. The EF calculations were modelled with that reported by Chen et al [41]. Assuming that immersion in 1 mM MBT would provide a dense monolayer of MBT on the surface of the Ag SERS substrate, which is estimated to be 4 MBT molecules/nm^2^ [41]. With a laser spot size of 12.68 µm for the 514 nm Raman spectrometer with a 20× objective (0.4 N.A.), the number of MBT molecules interrogated for SERS spectrum were estimated to be ~5.1 × 10^8^. The corresponding number of MBT molecules interrogated for normal Raman spectrum was estimated to be ~2.0 × 10^14^. This estimation was based on the theoretical volume of MBT molecule of about 300 Å^3^ [41], an illuminated area of diameter 12.68 µm and a laser penetration depth of about 484 µm at 5 mW laser power. The estimated enhancement factors for MBT on the different test Ag SERS substrates in this study were in the region of 10^5^ times (Table 4). Typical SER enhancement estimations fall in the range of 10^4^ to 10^8^ [38] and hence SERS enhancement factor greater than 10^5^ observed here for the non-resonant Raman active molecule, MBT, is comparable to that typically reported in literature.

Among the non-heated Ag substrates, the 30 s NH_4_OH + 10 s HNO_3_ treatment produced the highest SERS enhancement, while that by non-chemical etched native Ag substrate the lowest for the MBT peaks—1076 cm^−1^ and 1595 cm^−1^ (Figure 7a, Table 4). The surface roughness was lowest for 30 s NH_4_OH Ag substrate, but its surface topography was similar to that observed for native Ag substrate. Similarly, the SERS enhancement for NH_4_OH was comparable to that observed for Native Ag substrate. At the other end, 30 s NH_4_OH + 2 m HNO_3_ Ag substrate had the roughest surface. However, it showed the lowest SERS enhancement among the four different HNO_3_ treated samples, which can be attributed to the larger separation between roughness features on their surface. The 10 s HNO_3_ and the 30 s NH_4_OH + 10 s HNO_3_ Ag substrates showed a flaky morphology with narrow gaps between the flaky features compared to the 2 m HNO_3_ and the 30 s NH_4_OH + 2 m HNO_3_ Ag substrates. Thus, the highest SERS enhancements observed for both the MBT peaks, 1076 cm^−1^ and 1595 cm^−1^, suggests that 30 s NH_4_OH + 10 s HNO_3_ treatment produced Ag substrates having optimum separation between its surface roughness features.

Heat treatment at 130 °C for 1 h caused a decrease in SERS enhancements for each of the test Ag substrates compared to their respective non-heated counterparts (Figure 7b, Table 4). 30 s NH_4_OH + 10 s HNO_3_ Ag substrate again had the highest SERS enhancement. However, 30 s NH_4_OH + 2 m HNO_3_ showed the lowest SERS enhancement (Figure 7b, Table 4). The general decrease in SERS enhancements caused by heating, again, in conjunction with the general decrease in roughness parameters (R_t_, R_q_ and R_a_) further indicates that the height of the roughness features on the surface of the Ag substrates also plays an important role in the SERS signal intensities. This decreasing effect was maximum for 30 s NH_4_OH + 2 m HNO_3_ Ag substrate, suggesting that the longer HNO_3_ treatment duration potentially results in relatively fragile surface features susceptible to heat induced reorganization of surface roughness. On the other hand, the anomaly of increased roughness for 30 s NH_4_OH Ag substrate caused by heating, in turn, did not translate to increased SERS enhancement. This indicates that there not only exists an optimum spacing between the roughness features, but also an optimum height for realising the maximum SERS enhancement.

### 3.5. Quantitation of MBT Using SERs on 30 s NH_4_OH + 10 s HNO_3_ Ag Substrate

**Figure 8:**
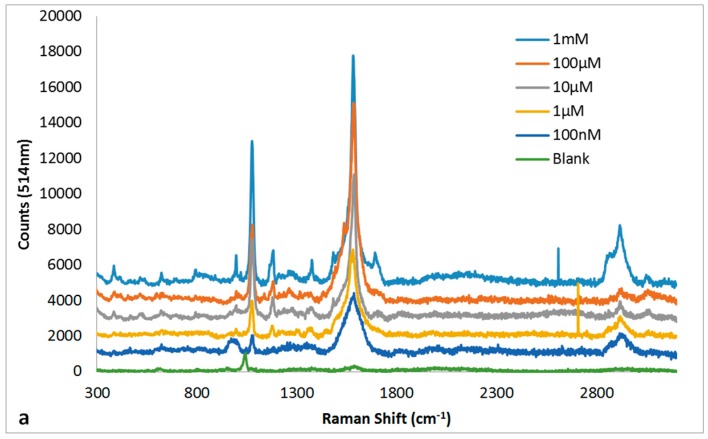

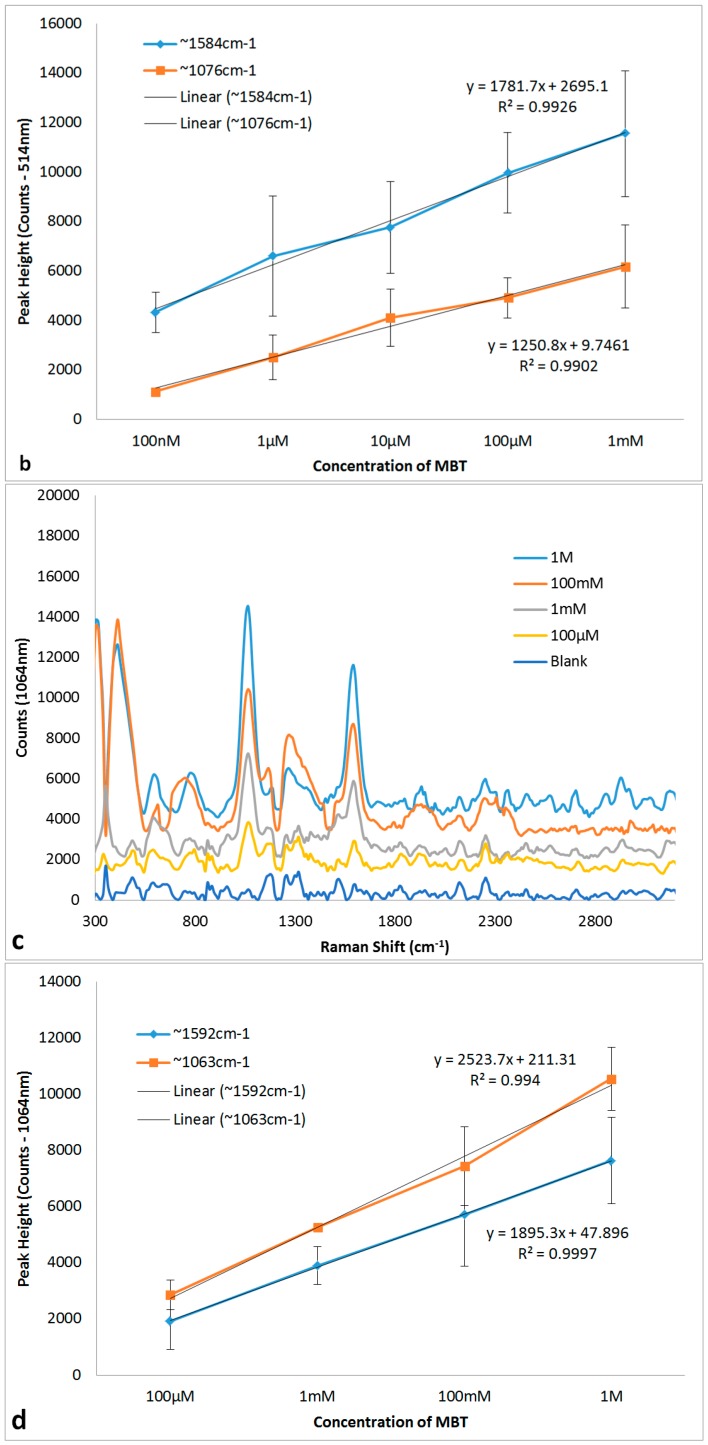
SERS spectra for MBT (**a**,**c**) as a function of concentration and their corresponding standard curves for the peaks ~1076 cm^−1^ and ~1584 cm^−1^ (**b**,**d**) recorded using 514 nm (**a**,**b**) and 1064 nm (**c**,**d**) Raman spectrometers. The SERS spectra (**a**,**c**) are offset by 1000 cm^−1^ for clarity. The peak heights (**b**,**d**) are presented as mean ± standard error of mean (*n* = 6).

The non-heated 30 s NH_4_OH + 10 s HNO_3_ Ag substrate that produced the best SERS enhancement was then carried forward for quantitation of MBT using both 514 nm and 1064 nm Raman spectrometers. The absolute intensities for the SERS peaks, ~1076 cm^−1^ and ~1595 cm^−1^, on both the 514 nm and 1064 nm SERS spectra (Figure 8a,c) for MBT as a function of concentration were used for plotting standard curves (Figure 8b,d) for quantitation of MBT. The linearity (R^2^ > 0.99) observed for SERS peak intensities for both ~1076 cm^−1^ and ~1595 cm^−1^ on both 514 nm and 1064 nm SERS spectra as a function of concentration was remarkable. Linear detection ranges tested, in this study, for quantitation of MBT were 100 nM to 1 mM using 514 nm Raman and 100 µM to 1 M using 1064 nm Raman. Regarding the limit of detection, the SERS peak intensities for both ~1076 cm^−1^ and ~1595 cm^−1^ peaks were above the background noise at concentrations ≥1 pM using 514 nm and ≥100 µM using 1064 nm Raman spectroscopy for MBT.

SERS enhancement efficacy is the fundamental property of a SERS substrate and Ag metal as a SERS substrate is known to provide the best SERS enhancement typically in the visible to near-infrared excitation [20,42]. In this study, we showed that chemically roughened solid Ag metal provides SERS enhancement even with the 1064 nm laser Raman spectrometer. Longer wavelength Raman spectroscopy requires higher laser powers to obtain meaningful Raman/SERS spectra. The higher laser power, in turn, causes heating and destruction of most SERS substrates [42]. The 1064 nm laser SERS spectra obtained with the chemically etched Ag substrate can be attributed to the ability of the solid Ag metal to dissipate the heat generated by the high power laser. From our experience (unpublished data), metal nanoparticles, nano-patterned polymer substrates coated with metal or metal nanoparticles’ coated filter papers all fail to provide SERS spectra due to destruction of the substrate by the high power 1064 nm laser beam. Thus, the heat dissipation ability of Ag metal can potentially open up the use of longer wavelength Raman spectroscopy for SERS-based detection. Such broadband SERS substrates would be beneficial for a universal lab-on-a-chip device [43].

## 4. Conclusions

In this study, we showed the efficacy of passive chemical etching with NH_4_OH and/or HNO_3_ on generating roughened surface on solid Ag metal for SERS detection using 514 nm and 1064 nm Raman spectrometers. MBT (p-thiocresol), a Raman active molecule, was used to assess SERS efficacy of these substrates. The effect of heating on surface roughness and SERS efficacy of the native and chemical etched Ag substrates was also investigated. Etching with 35% NH_4_OH for 30 s resulted in smoothening of the Ag surface, whilst etching for 10 s or 2 m with 6 M HNO_3_ produced a much rougher surface. Synergistic effect of the smoothening by 30 s NH_4_OH treatment followed by 10 s or 2 m etching by HNO_3_ produced uniformly distributed surface roughness features. Quantitative interferometry results showed microscale roughness (maximum peak to valley distance ranging between 10 and 15 µm) on all test Ag substrates. The 30 s NH_4_OH + 2 m HNO_3_ treatment produced the roughest surface (more peaks and valleys in conjunction with larger peak heights and deeper valleys from the mean linear surface). However, the surface with comparatively lower roughness generated by 30 s NH_4_OH + 10 s HNO_3_ treatment produced the maximum SERS enhancement for the Ag substrate indicating that there is an optimum height of and spacing between the surface roughness features wherein SERS enhancement is maximised. Further proof that optimum spacing and height of the surface roughness features determine the degree of SERS enhancement is observed with heat treatment of the different Ag substrates. Heating caused a restructuring of the surface roughness features resulting in a general decrease not only in the surface roughness, but also in the SERS enhancement for all test Ag substrates. The 30 s NH_4_OH + 10 s HNO_3_ Ag substrate that gave the best SERS enhancement was then tested for efficacy in quantifying MBT using 514 nm and 1064 nm Raman spectrometers. Standard curves with R^2^ > 0.99 and limits of detection of 1 pM and 100 µM respectively on 514 nm and 1064 nm SERS spectra were obtained using absolute intensities for the typical SERS peaks at ~1076 cm^−1^ and ~1595 cm^−1^ of MBT. Our results clearly demonstrate the rapid and the synergistic effect of ammonia and nitric acid treatments in generating a robust broadband SERS substrate on solid Ag metal surface for SERS detection and quantitation. Of particular interest is the ability of solid Ag metal in generating reliable SERS enhancements through dissipation of heat generated by high power lasers (e.g., 1064 nm) used in longer wavelength Raman spectroscopy.

## Figures and Tables

**Table 1:** Surface (area) roughness data for the native flattened Ag wire and the different flattened Ag wires after chemical etching treatments. (*n* = 6, mean ± standard error of mean).

	R_t_ (PV) (µm)	R_p_ (µm)	R_v_ (µm)	R_q_ (RMS) (µm)	R_a_ (µm)	(R_q_−R_a_)/R_a_ (%)
Native	11.15 ± 0.89	5.83 ± 0.64	5.31 ± 0.53	1.27 ± 0.17	0.95 ± 0.10	32.20
30 s NH_4_OH	12.04 ± 1.15	5.99 ± 0.84	6.06 ± 0.67	1.02 ± 0.19	0.77 ± 0.15	31.90
10 s HNO_3_	14.63 ± 2.09	6.48 ± 1.01	8.15 ± 1.10	1.33 ± 0.27	1.03 ± 0.21	28.96
2 m HNO_3_	10.04 ± 0.59	4.97 ± 0.39	5.06 ± 0.23	1.23 ± 0.10	0.96 ± 0.09	27.51
30 s NH_4_OH + 10 s HNO_3_	13.09 ± 0.98	6.05 ± 0.58	7.03 ± 0.52	1.40 ± 0.30	1.05 ± 0.21	33.52
30 s NH_4_OH + 2 m HNO_3_	14.08 ± 1.92	5.98 ± 0.98	8.10 ± 1.10	1.95 ± 0.41	1.39 ± 0.30	40.08

**Table 2:** Surface (area) roughness data for the native flattened Ag wire and the different flattened Ag wires after chemical etching treatments. (*n* = 6, mean ± standard error of mean).

	R_t_ (PV) (µm)	R_p_ (µm)	R_v_ (µm)	R_q_ (RMS) (µm)	R_a_ (µm)	(R_q_−R_a_)/R_a_ (%)
Native	9.59 ± 0.75	4.39 ± 0.25	5.20 ± 0.59	1.20 ± 0.11	0.93 ± 0.08	29.18
30 s NH_4_OH	12.99 ± 1.33	7.12 ± 0.59	5.87 ± 0.93	1.53 ± 0.15	1.19 ± 0.12	28.62
10 s HNO_3_	14.79 ± 1.17	6.88 ± 0.97	7.90 ± 0.35	1.29 ± 0.04	1.00 ± 0.03	29.75
2 m HNO_3_	11.69 ± 1.94	6.02 ± 1.33	5.67 ± 0.80	1.35 ± 0.23	1.07 ± 0.18	26.30
30 s NH_4_OH + 10 s HNO_3_	15.18 ± 1.55	7.23 ± 0.86	7.95 ± 0.89	1.58 ± 0.19	1.19 ± 0.14	32.66
30 s NH_4_OH + 2 m HNO_3_	12.69 ± 0.48	5.67 ± 0.40	7.02 ± 0.25	1.40 ± 0.11	1.09 ± 0.08	29.11

**Table 3:** Raman and SERS peaks (cm^−1^) for MBT on spectra recorded using 514 nm and 1064 nm Raman spectrometers and the peak assignments.

514 nm Raman Peaks (cm^−1^)		1064 nm Raman Peaks (cm^−1^)		Peak Assignment
Raman Spectrum	SERS Spectrum	Raman Spectrum	SERS Spectrum	
312	314			τ_r_CC [35]
377	388	347	353	δ_r_CC [34,36]
484				γ_r_CC [34,36]
634	623	609	608	υCS [34]
794	791	781		τ_r_CH [34]
913	995, 1014			γ_r_CH [34]
1097	1076	1085	1063	υCS [34,35,36,37,38]
1184	1180	1198		υ_r_CC [34]
1209	1258			υ_r_CC [35]
				υCH_3_ [35]
1309, 1374	1377	1372		υCH_3_ [34,35]
1591	1483, 1584	1592	1592	υ_r_CC [34,35,36,37,38]
2562		2573		υSH [34,39]
2727		2739		δCH_3_ [34,40]
2855, 2914	2919	2918		υ_s_CH_3_ [34,40]
2978				υ_as_CH_3_ [34,40]
3036, 3056		3059		υ_r_CH [34,40]

υ—stretching, δ—in-plane bending, γ—out-of-plane bending, τ—torsion, r—ring, s—symmetric, as—asymmetric.

**Table 4:** SERS enhancements for the different test Ag substrates, in this study: As illustrated by the absolute intensities (AI, counts ± standard error of mean, *n* = 4) and enhancement factors (EF × 10^5^) for the ~1076 cm^−1^ and the ~1595 cm^−1^ SERS peaks for 1 mM MBT as a function of chemical etching, with and without heat treatments using 514 nm laser wavelength.

	1076 cm^−1^				1595 cm^−1^			
	Non-Heated		Heated		Non-Heated		Heated	
	AI Counts	EF × 10^5^	AI Counts	EF × 10^5^	AI Counts	EF × 10^5^	AI Counts	EF × 10^5^
**Native Ag Substrate**	1728 ± 629	3.3	1209 ± 901	2.3	1883 ± 779	5.3	1087 ± 957	3.0
**30 s NH_4_OH**	2104 ± 266	4.0	933 ± 414	1.8	1630 ± 126	4.6	815 ± 283	2.3
**10 s HNO_3_**	3506 ± 446	6.6	2352 ± 330	4.5	3656 ± 584	10.2	1876 ± 357	5.2
**2 m HNO_3_**	1035 ± 336	2.0	1884 ± 337	3.6	4672 ± 622	13.1	1563 ± 362	4.4
**30 s NH_4_OH + 10 s HNO_3_**	5825 ± 849	11.0	2093 ± 549	4.0	6027 ± 989	16.9	3166 ± 964	8.9
**30 s NH_4_OH + 2 m HNO_3_**	1994 ± 495	3.8	434 ± 109	0.8	2257 ± 540	6.3	476 ± 92	1.3
